# Body iron stores and risk of type 2 diabetes: results from the European Prospective Investigation into Cancer and Nutrition (EPIC)-Potsdam study

**DOI:** 10.1007/s00125-012-2633-y

**Published:** 2012-07-01

**Authors:** J. Montonen, H. Boeing, A. Steffen, R. Lehmann, A. Fritsche, H.-G. Joost, M. B. Schulze, T. Pischon

**Affiliations:** 1 Department of Epidemiology, German Institute of Human Nutrition Potsdam-Rehbruecke, Arthur-Scheunert-Allee 114-116, 14558 Nuthetal, Germany; 2 Central Laboratory/Clinical Chemistry and Pathobiochemistry, University Hospital Tübingen, Tübingen, Germany; 3 Paul Langerhans Institute Tübingen (Inst. for Diabetes Research and Metabolic Diseases of the Helmholtz Centre Munich at the University of Tübingen), Tübingen, Germany; 4 Department of Internal Medicine IV, University of Tübingen, Tübingen, Germany; 5 Department of Pharmacology, German Institute of Human Nutrition Potsdam-Rehbruecke, Nuthetal, Germany; 6 Department of Molecular Epidemiology, German Institute of Human Nutrition Potsdam-Rehbruecke, Nuthetal, Germany; 7 Molecular Epidemiology Group, Max Delbrück Center for Molecular Medicine (MDC), Berlin-Buch, Germany

**Keywords:** Body iron store, Ferritin, Soluble transferrin receptor, Type 2 diabetes

## Abstract

**Aims/hypothesis:**

The aim of this study was to prospectively examine the association between body iron stores and risk of type 2 diabetes.

**Methods:**

We designed a case–cohort study among 27,548 individuals within the population-based European Prospective Investigation into Cancer and Nutrition (EPIC)-Potsdam study. During 7 years of follow-up, 849 incident cases of type 2 diabetes were identified. Of these, 607 remained for analyses after exclusion of participants with missing data or abnormal glucose levels at baseline. A sub-cohort of 2,500 individuals was randomly selected from the full cohort, comprising 1,969 individuals after applying the same exclusion criteria.

**Results:**

After adjustment for age, sex, BMI, waist circumference, sports activity, bicycling, education, occupational activity, smoking habit, alcohol consumption and circulating levels of γ-glutamyltransferase, alanine aminotransferase, fetuin-A, high-sensitivity C-reactive protein, adiponectin, HDL-cholesterol and triacylglycerol, higher serum ferritin concentrations were associated with a higher risk of type 2 diabetes (RR in the highest vs lowest quintile, 1.73; 95% CI 1.15, 2.61; *p*_trend_ = 0.002). No significant association was observed for soluble transferrin receptor (RR 1.33; 95% CI 0.85, 2.09; *p*_trend_ = 0.50). The soluble transferrin receptor-to-ferritin ratio was significantly inversely related to risk (RR 0.61; 95% CI 0.41, 0.91; *p*_trend_ = 0.02).

**Conclusions/interpretation:**

High ferritin levels are associated with higher risk of type 2 diabetes independently of established diabetes risk factors and a range of diabetes biomarkers whereas soluble transferrin receptor concentrations are not related to risk. These results support the hypothesis that higher iron stores below the level of haemochromatosis are associated with risk of type 2 diabetes.

## Introduction

Impaired glucose metabolism and diabetes mellitus are common clinical manifestations of iron overload in patients with haemochromatosis [1], a disorder of abnormal iron absorption resulting in the progressive accumulation of iron in inner organs. The precise molecular mechanisms underlying the pathogenesis of iron-overload-related diabetes have not been identified but the initial glucose abnormalities include insulin resistance and hyperinsulinaemia, followed by impaired insulin secretion [2]. Elevated iron stores below the levels of known iron overload syndromes have also been implicated in the aetiology of diabetes [2, 3]. However, so far only a few studies have prospectively examined the association between body iron stores and risk of diabetes [4–7].

Serum ferritin concentration is by far the most commonly used indicator of body iron stores in epidemiological studies. The use of serum ferritin in assessing iron stores, however, is complicated because ferritin is also an acute-phase protein that may be elevated in inflammation, liver disease and cancer [8]. Sub-clinical inflammation, which is usually observed in individuals with obesity and the metabolic syndrome, is associated with increased risk of type 2 diabetes [9]. It is thus unclear whether the association of ferritin with type 2 diabetes reflects these other abnormalities. Circulating ferritin levels have also been shown to be positively associated with circulating level of hepatic enzymes [4, 10] and markers of dyslipidaemia [11, 12], and negatively associated with adiponectin levels [4]. Thus, in a recent nested case–control study the association observed between serum ferritin and diabetes risk disappeared after adjustment for components of the metabolic syndrome [13].

Furthermore, serum ferritin concentrations may not be an accurate reflection of the intracellular iron pool, which is likely to be responsible for iron-related oxidative stress [14]. Serum soluble transferrin receptor (sTfR) concentration has been suggested as a more accurate measure of available body iron [15, 16]. The level of circulating sTfR reflects the availability of iron in the body and it can be used in the clinical setting to distinguish between iron-deficiency anaemia and anaemia due to chronic diseases [16]. When the iron–transferrin complex binds to its cellular receptor (TfR), iron enters cells and the soluble extracellular domain of TfR is liberated into the circulation [17]. Serum levels of this soluble form (sTfR) are therefore directly proportional to tissue TfR concentration. Circulating sTfR levels correlate inversely with body iron stores and thus reflect, inversely, intracellular iron storage, and the ratio of sTfR to ferritin has been proposed as a preferred measure of body iron stores to quantifiably reflect body iron over the entire spectrum of iron balance [18, 19].

The objective of this study was to examine prospectively the association of body iron stores, as assessed by circulating ferritin concentrations and sTfR, as well as by the ratio of sTfR to ferritin, with risk of incident type 2 diabetes in a population-based cohort. We also investigated whether this association is independent of inflammation as well as markers of different metabolic pathways that may be involved in the pathogenesis of type 2 diabetes.

## Methods

### Study population

The EPIC-Potsdam study is part of the multicentre European Prospective Investigation into Cancer and Nutrition (EPIC) [20, 21]. In Potsdam, Germany, 27,548 individuals (16,644 women mainly aged 35–65 years and 10,904 men mainly aged 40–65 years) were recruited from the general population between 1994 and 1998 [22]. The baseline examination included anthropometric measurements, a personal health interview, a health questionnaire and blood sampling. Informed consent was obtained from participants; approval was given by the Ethics Committee of the Medical Association of the State of Brandenburg, Germany.

The health interview and the questionnaire elicited information on educational attainment, smoking and alcohol consumption habits, and occupational and leisure-time physical activity. The latter was assessed as sports activity and cycling, both calculated as the average time spent per week during the 12 months before recruitment. Waist circumference was measured midway between the lower rib margin and the superior anterior iliac spine to the nearest 0.5 cm with a non-stretching tape applied horizontally [23]. Weight and height were measured without shoes in light clothing and BMI was calculated as kg/m^2^.

The prevalence of diabetes at baseline was evaluated by a physician using information on self-reported medical diagnoses, medication records and ongoing nutritional therapy. Uncertainties regarding a proper diagnosis were clarified with the participant or their physician.

Incident cases of diabetes were identified during follow-up until 31 August 2005 via self-reports of a diabetes diagnosis, diabetes-relevant medication or dietary treatment related to diabetes. All incident cases were verified by questionnaires mailed to the diagnosing physician, requesting details of the date and type of diagnosis, diagnostic tests and treatment of diabetes. Only individuals with a diagnosis of type 2 diabetes that was confirmed by a physician (ICD-10; www.who.int/classifications/icd/en/) and a diagnosis date after the baseline examination were considered as incident cases of type 2 diabetes.

Our study was conducted in a prospective case–cohort design [24]. For this, we selected a sub-cohort of 2,500 individuals from all participants in the EPIC-Potsdam study who provided blood samples at baseline (*n* = 26,444). Of these, 1,969 participants remained for analyses after excluding participants with any history of diabetes at baseline or with self-reported but unverified diabetes during follow-up, with missing values for study variables or with abnormal plasma glucose (random glucose 11.1 mmol/l or more, or fasting glucose 7.0 mmol/l or more, or less than 2.8 mmol/l). By randomly selecting a sub-cohort and using appropriate statistics for this research design, the results are expected to be generalisable to the entire cohort without the need of biomarker measurements in the entire EPIC-Potsdam study. During an average follow-up time of 7 years, a total of 849 incident cases of type 2 diabetes were identified in the full cohort. Of these, 607 remained for analyses after applying similar exclusion criteria. In agreement with the case–cohort design, 56 individuals who developed type 2 diabetes during follow-up were identified in the sub-cohort (so called internal cases [24]), while 551 incident cases were identified outside the sub-cohort (external cases).

### Clinical and biomarker measurements

A total of 30 ml of venous blood was collected at baseline from participants; it was fractionated into serum, plasma, buffy coat and erythrocytes and was liquated into straws and stored in liquid nitrogen at 170°C for conservation until the time of analysis. Serum and plasma samples were transferred to the Department of Internal Medicine, University of Tübingen, on dry ice for analyses. Serum ferritin concentrations were determined using an ADVIA Centaur XP and serum sTfR concentrations using a BN ProSpec System (both Siemens Healthcare, Erlangen, Germany). Plasma levels of alanine aminotransferase (ALT), γ-glutamyltransferase (GGT), HDL-cholesterol, triacylglycerol and high-sensitivity C-reactive protein (hs-CRP) were determined using the automatic ADVIA 1650 analyser (Siemens Medical Solutions, Erlangen, Germany). Total adiponectin concentration was measured using an ELISA (Linco Research, St Charles, MI, USA). For determination of fetuin-A an immunoturbidimetric method was used with specific polyclonal goat anti-human fetuin-A antibodies to human fetuin-A (BioVendor Laboratory Medicine, Modreci, Czech Republic) [25]. Erythrocyte levels of HbA_1c_ were measured using an HPLC procedure according to the manufacturer's instructions (Tosoh, Stuttgart, Germany). Total adiponectin concentration was measured using an ELISA (Linco Research). All assay procedures were performed as described by the manufacturer.

### Statistical analysis

The correlation between biomarkers of body iron stores and diabetes risk factors was studied in the sub-cohort with age- and sex-adjusted partial Pearson correlation coefficients. Skewed variables were log-transformed. Circulating levels of ferritin, sTfR and the ratio of sTfR to ferritin were categorised into sex-specific quintiles based on sub-cohort participants. The RR of type 2 diabetes with 95% CI for quintiles of measures of body iron stores was calculated using the proportional hazards model, modified for the case–cohort design according to the Prentice method [24]. Age at baseline in 1-year categories was entered as a stratum variable. Age was also used as an underlying time variable in the counting processes, with entry defined as the participant’s age at recruitment and exit defined as age at diabetes diagnosis or censoring. Models were adjusted for sex, BMI (continuous), waist circumference (continuous), education (in or no training, vocational training, technical school, or technical college or university degree), occupational activity (light, moderate or heavy), sports activity (0, 0.1–4 or over 4 h/week), cycling (0, 0.1–2.4, 2.5–4.9 or 5 or more h/week), smoking habit (never, past, current ≤20 cigarettes/day or current >20 cigarettes/day) and alcohol intake (0, 0.1–5, 5.1–10, 10.1–20, 20.1–40 or over 40 g/day). In further models, we additionally adjusted for metabolic biomarkers (hs-CRP; GTT and ALT; HDL-cholesterol and triacylglycerol; and adiponectin, all continuous). In addition, a model including energy intake and consumption of coffee, red meat and whole-grain bread was used. In additional analyses we excluded individuals (*n* = 46) in whom serum ferritin was elevated beyond three times the SD from the mean (25 women with ferritin over 37.3 mg/l, 21 men with ferritin over 100.0 mg/l) to reduce the possibility of including those with undiagnosed obvious haemochromatosis. Tests of linear trends in RR across quintiles of the biomarker levels were carried out by including the quintile of iron store variable in the model as a continuous variable. Since no evidence of a statistical interaction between measures of body iron and sex was observed in a model including an interaction term, the analyses were performed in men and women combined (*p* for interaction 0.80, 0.24 and 0.69 for ferritin, sTfR and the ratio of sTfR to ferritin, respectively).

All *p* values presented are two-tailed, and *p* < 0.05 was considered statistically significant. All analyses were performed using SAS software (version 9.1; SAS Institute Inc, Cary, NC, USA).

## Results

Baseline characteristics across quintiles of ferritin in the random sub-cohort are presented in Table 1. Participants with higher ferritin concentrations tended to be older, more inactive and have a higher BMI and waist circumference, and they reported higher alcohol consumption compared with participants who had lower ferritin concentrations. Consumption of red meat and coffee increased across quintiles of serum ferritin, while consumption of whole-grain bread decreased. Concentrations of the hepatic enzymes ALT and GGT, as well as hs-CRP and triacylglycerol, were higher with increasing ferritin categories, whereas adiponectin and HDL-cholesterol concentrations were lower.

**Table 1:** Characteristics of study participants according to quintiles of plasma ferritin level in the sub-cohort

Characteristic	Quintiles of plasma ferritin^a^				
	1 (*n* = 377)	2 (*n* = 394)	3 (*n* = 389)	4 (*n* = 398)	5 (*n* = 411)
Age (years), mean (SD)	47.1 (8.74)	48.1 (8.73)	48.9 (8.86)	51.4 (8.63)	54.6 (8.16)
Sex (% men)	39.3	34.0	36.5	40.5	38.2
Current or former smoker (%)	54.4	53.8	50.4	50.0	52.1
University degree education (%)	40.6	37.8	42.4	37.7	31.1
BMI (kg/m^2^), mean (SD)	24.7 (3.59)	25.0 (4.09)	26.1 (3.97)	26.2 (4.15)	27.8 (4.51)
Waist circumference (cm), mean (SD)	81.8 (11.46)	82.2 (12.3)	85.5 (12.4)	86.3 (11.9)	90.6 (12.8)
Alcohol consumption (g/day), mean (SD)	13.1 (16.18)	11.2 (12.5)	13.6 (19.9)	15.1 (17.9)	17.0 (29.1)
Sports activity (h/week), mean (SD)	1.33 (2.26)	1.05 (1.64)	1.04 (1.76)	1.03 (1.79)	0.69 (1.29)
Bicycling (h/week), mean (SD)	1.87 (2.73)	1.76 (2.89)	1.48 (2.22)	1.99 (2.88)	1.86 (3.06)
Heavy or very heavy work strain (%)	5.84	5.58	5.40	6.03	8.03
Energy intake (kJ/day), mean (SD)	8,986 (2847)	8,642 (2731)	8,543 (2506)	8,647 (2688)	8,459 (2874)
Red meat (servings/day), mean (SD)	0.26 (0.20)	0.28 (0.19)	0.28 (0.19)	0.29 (0.18)	0.30 (0.20)
Whole-grain bread (servings/day), mean (SD)	0.96 (1.03)	0.96 (1.05)	0.97 (1.14)	0.94 (1.15)	0.77 (0.98)
Coffee (cups/day), mean (SD)	2.78 (2.39)	2.74 (1.82)	2.75 (2.12)	2.86 (2.15)	2.81 (2.00)
Plasma levels, geometric mean (95% CI)					
sTfR (mg/l)	1.09 (1.06, 1.12)	1.00 (0.98, 1.02)	0.98 (0.96, 1.00)	1.00 (0.98, 1.02)	1.02 (1.00, 1.04)
sTfR/ferritin ratio	0.58 (0.53, 0.64)	0.23 (0.22, 0.25)	0.13 (0.12, 0.14)	0.08 (0.08, 0.09)	0.04 (0.04, 0.05)
Adiponectin (ng/ml)	7.39 (7.05, 7.76)	7.26 (6.92, 7.62)	7.30 (6.94, 7.67)	7.15 (6.81, 7.51)	7.02 (6.68, 7.38)
GGT (U/l)	14.1 (13.0, 15.2)	14.7 (13.7, 15.7)	18.6 (17.1, 20.3)	19.3 (17.9, 20.9)	25.4 (23.3, 27.8)
ALT (U/l)	17.2 (16.5, 18.0)	17.7 (16.9, 18.5)	19.4 (18.5, 20.4)	19.6 (18.7, 20.5)	24.1 (22.8, 25.5)
Fetuin-A (μg/ml)	250 (245, 256)	247 (240, 253)	245 (238, 253)	243 (238, 249)	237 (232, 243)
hs-CRP (mg/l)	0.52 (0.45, 0.59)	0.55 (0.48, 0.63)	0.80 (0.70, 0.90)	0.87 (0.76, 0.99)	1.02 (0.89, 1.16)
HDL-cholesterol (mmol/l)	1.34 (1.31, 1.38)	1.35 (1.32, 1.39)	1.32 (1.28, 1.36)	1.29 (1.26, 1.32)	1.26 (1.23, 1.30)
Triacylglycerol (mmol/l)	1.07 (1.01, 1.13)	1.02 (0.97, 1.08)	1.17 (1.11, 1.23)	1.26 (1.19, 1.34)	1.40 (1.32, 1.48)

^a^Ferritin quintiles for men were <8.00, 8.00 to <13.0, 13.0 to <19.0, 19.0 to <28.0 and ≥28.0 mg/l; and for women ferritin quintiles were <2.00, 2.00 to <3.89, 3.89 to <6.39, 6.39 to <11.0, and ≥11.0 mg/l

The markers of iron stores were weakly to moderately correlated with other metabolic biomarkers and with BMI and waist circumference (Table 2). For example, the age- and sex-adjusted partial Pearson coefficients for the correlations of waist circumference with ferritin, sTfR and sTfR-to-ferritin ratio were 0.23 (*p* < 0.001), 0.11 (*p* < 0.001) and −0.18 (*p* < 0.001), respectively.

**Table 2:** Age and sex adjusted partial Pearson correlation coefficients between serum levels^a^ of iron markers and biomarkers of selected risk factors of diabetes in the sub-cohort

	Ferritin		sTfR		sTfR/ferritin ratio	
Marker or biomarker	*r*	*p* value	*r*	*p* value	*r*	*p* value
sTfR	−0.16	<0.001				
sTfR/ferritin ratio	−0.97	<0.001	0.41	<0.001		
BMI	0.21	<0.001	0.10	<0.001	−0.17	<0.001
Waist circumference	0.23	<0.001	0.11	<0.001	−0.18	<0.001
Red meat intake	0.09	<0.001	0.01	0.51	−0.08	<0.001
Adiponectin	−0.08	<0.001	0.02	0.32	0.08	<0.001
GGT	0.26	<0.001	0.08	<0.001	−0.22	<0.001
ALT	0.24	<0.001	0.07	0.003	−0.21	<0.001
Fetuin-A	−0.04	0.07	0.08	<0.001	0.06	0.01
hs-CRP	0.16	<0.001	0.04	0.11	−0.14	<0.001
HDL-cholesterol	−0.08	<0.001	−0.03	0.14	0.07	0.003
Triacylglycerol	0.13	<0.001	0.03	0.20	−0.15	<0.001

No. of individuals included in sub-cohort, *n* = 1,969

^a^Based on log-transformed values of biomarkers

A significant association was observed between serum ferritin concentration and type 2 diabetes risk (Table 3). The relative risk in the highest compared with the lowest quintile of ferritin was 2.00 (95% CI 1.35, 2.95; *p*_trend_< 0.001) when adjusted for age, sex, BMI, waist circumference, sports activity, bicycling, education, occupational activity, smoking habit and alcohol consumption. We next examined the impact of adjustment for different biomarkers on the association of measures of iron status with risk of type 2 diabetes. In these analyses, adjustment for GGT and ALT tended to have the strongest effect in terms of attenuating the risk for the association of ferritin with risk of diabetes, whereas adjustment for hs-CRP tended to have a weaker effect. Results remained virtually unchanged when hs-CRP was adjusted for in established risk categories (<1, 1–3, ≥3 mg/l) [26] instead of using a linear term. Although mutual adjustment for hs-CRP, GGT, ALT, adiponectin, HDL-cholesterol and triacylglycerol further attenuated the association, it still remained significant. Specifically, the RR for the extreme quintile of ferritin was 1.60 (95% CI 1.07, 2.41; *p*_trend_ = 0.007). In contrast to ferritin, no significant association was observed between serum levels of sTfR and type 2 diabetes risk. The corresponding multivariate RR for the highest vs lowest quintile of sTfR was 1.22 (95% CI 0.81, 1.86; *p*_trend_ = 0.64). Further adjustment for ferritin did not notably alter the result (data not shown). Finally, a significant inverse association was observed between sTfR-to-ferritin ratio and type 2 diabetes risk in models adjusting for various lifestyle factors (RR 0.50; 95% CI 0.34, 0.73; *p*_trend_ < 0.001). Only marginal changes in risk estimates were observed in models further adjusting for different biomarkers. In a final model including markers of inflammation and dyslipidaemia, as well as adiponectin and liver enzymes, the RR for extreme quintiles of sTfR-to-ferritin ratio was 0.61 (95% CI 0.41, 0.91; *p*_trend_ = 0.02). Further adjustment for dietary factors, including energy intake, consumption of red and processed meat, coffee, whole-grain bread, magnesium and dietary iron did not notably alter the associations observed for ferritin and sTfR-to-ferritin ratio (data not shown); neither did further adjustment for circulating HbA_1c_ level (data not shown).

**Table 3:** RR of type 2 diabetes (95% CI) according to serum ferritin, sTfR and the ratio of sTfR to ferritin

Risk factor	RR (95% CI)					*p*_trend_
	Quintile 1	Quintile 2	Quintile 3	Quintile 4	Quintile 5	
Ferritin						
Men, mg/l	<8.00	8.00 to <13.0	13.0 to <19.0	19.0 to <28.0	≥28.0	
Women, mg/l	<2.00	2.00 to <3.89	3.89 to <6.39	6.39 to <11.0	≥11.0	
Model 1	1	0.95 (0.59, 1.50)	1.49 (1.00, 2.24)	1.54 (1.03, 2.31)	2.00 (1.35, 2.95)	<0.001
Model 1 + hs-CRP	1	1.01 (0.63, 1.63)	1.56 (1.03, 2.37)	1.65 (1.09, 2.50)	2.09 (1.40, 3.12)	<0.001
Model 1 + GGT and ALT	1	0.91 (0.57, 1.46)	1.45 (0.97, 2.17)	1.51 (1.01, 2.26)	1.74 (1.17, 2.60)	<0.001
Model 1 + fetuin-A	1	0.94 (0.59, 1.51)	1.53 (1.01, 2.30)	1.58 (1.05, 2.38)	2.13 (1.43, 3.16)	<0.001
Model 1 + adiponectin	1	0.92 (0.57, 1.49)	1.52 (1.00, 2.30)	1.55 (1.03, 2.33)	1.95 (1.31, 2.90)	<0.001
Model 1 + HDL-cholesterol, triacylglycerol	1	1.00 (0.62, 1.61)	1.49 (0.99, 2.25)	1.49 (0.99, 2.23)	1.80 (1.22, 2.66)	<0.001
Model 2	1	1.00 (0.61, 1.65)	1.49 (0.97, 2.30)	1.59 (1.05, 2.42)	1.73 (1.15, 2.61)	0.002
sTfR						
Men, mg/l	<0.90	0.90 to <1.00	1.00 to <1.10	1.10 to <1.30	≥1.30	
Women, mg/l	<0.80	0.80 to <0.89	0.89 to <1.00	1.00 to <1.20	≥1.20	
Model 1	1	1.37 (0.89, 2.12)	1.83 (1.20, 2.79)	1.52 (1.03, 2.24)	1.22 (0.81, 1.86)	0.64
Model 1 + hs-CRP	1	1.42 (0.92, 2.19)	1.87 (1.23, 2.85)	1.51 (1.02, 2.23)	1.18 (0.78, 1.80)	0.85
Model 1 + GGT and ALT	1	1.33 (0.86, 2.07)	1.81 (1.19, 2.76)	1.46 (0.99, 2.16)	1.19 (0.78, 1.81)	0.75
Model 1 + fetuin-A	1	1.36 (0.88, 2.10)	1.78 (1.17, 2.71)	1.48 (1.00, 2.18)	1.14 (0.75, 1.73)	0.94
Model 1 + adiponectin	1	1.40 (0.89, 2.20)	1.81 (1.17, 2.81)	1.63 (1.09, 2.45)	1.30 (0.84, 2.00)	0.38
Model 1 + HDL-cholesterol, triacylglycerol	1	1.47 (0.94, 2.30)	1.93 (1.25, 2.97)	1.50 (1.00, 2.23)	1.36 (0.89, 2.08)	0.47
Model 2	1	1.38 (0.86, 2.20)	1.87 (1.20, 2.93)	1.47 (0.97, 2.22)	1.21 (0.78, 1.89)	0.80
sTfR-to-ferritin ratio						
Men, (mg/l)/(mg/l)	<0.04	0.04 to <0.05	0.05 to <0.08	0.08 to <0.13	≥0.13	
Women, (mg/l)/(mg/l)	<0.09	0.09 to <0.15	0.15 to <0.25	0.25 to <0.50	≥0.50	
Model 1	1	0.66 (0.48, 0.90)	0.59 (0.43, 0.82)	0.63 (0.45, 0.88)	0.50 (0.34, 0.73)	<0.001
Model 1 + hs-CRP	1	0.67 (0.49, 0.91)	0.59 (0.42, 0.82)	0.63 (0.45, 0.89)	0.47 (0.32, 0.70)	<0.001
Model 1 + GGT and ALT	1	0.72 (0.52, 0.99)	0.66 (0.47, 0.91)	0.69 (0.49, 0.98)	0.56 (0.38, 0.83)	0.004
Model 1 + fetuin-A	1	0.64 (0.46, 0.87)	0.57 (0.41, 0.79)	0.58 (0.41, 0.82)	0.46 (0.32, 0.68)	<0.001
Model 1 + adiponectin	1	0.71 (0.51, 0.97)	0.62 (0.44, 0.87)	0.64 (0.45, 0.92)	0.52 (0.35, 0.76)	<0.001
Model 1 + HDL-cholesterol, triacylglycerol	1	0.69 (0.50, 0.95)	0.64 (0.46, 0.89)	0.71 (0.50, 1.00)	0.55 (0.37, 0.80)	0.002
Model 2	1	0.76 (0.54, 1.06)	0.67 (0.47, 0.96)	0.70 (0.48, 1.02)	0.57 (0.38, 0.85)	0.005

Model 1 is adjusted for age, sex, BMI, waist circumference, sports activity, bicycling, education, occupational activity, smoking habit and alcohol consumption

Model 2 is adjusted for factors in model 1 and GGT, ALT, fetuin-A, hs-CRP, adiponectin, HDL-cholesterol and triacylglycerol concentrations

When we excluded 46 individuals with serum ferritin levels greater than or equal to three times the SD from the mean, no notable alteration in the results was observed (data not shown). Results were also not substantially different when we excluded 157 cases of type 2 diabetes that occurred within the first 2 years of follow-up (to further reduce the possibility of including individuals with undiagnosed diabetes at baseline) (data not shown). In further exploratory analyses we investigated potential effect modification of HbA_1c_ level, BMI, hs-CRP level or hypertension. We found a significant interaction for circulating HbA_1c_ level on the association between ferritin and diabetes risk (*p*_interaction_<0.001). Thus, the association observed between serum ferritin level and diabetes risk was significant among persons with HbA_1c_ of 5.3% (34.4 mmol/mol) or more (RR for the highest quintile 1.70; 95% CI 1.03, 2.80), but not among persons with HbA_1c_ values below 5.3% (34.4 mmol/mol) (RR for the highest quintile 0.97; CI 0.38, 2.50). Circulating HbA_1c_ also tended to modify the association between sTfR and diabetes risk, albeit the interaction term did not reach the significance level of 0.05 (*p*_interaction_ = 0.08). Thus, among participants with an HbA_1c_ level above 5.3% (34.4 mmol/mol), the RR for the highest vs lowest quintile was 1.15 (95% CI 0.69, 1.91), whereas for HbA_1c_ levels below 5.3% (34.4 mmol/mol) it was 2.39 (95% CI 0.79, 7.23). We found no significant interaction of ferritin, sTfR and the sTfR-to-ferritin ratio with BMI, hs-CRP level or hypertension, on risk of type 2 diabetes (data not shown).

## Discussion

In the present case–cohort study, higher body iron stores (reflected by elevated ferritin concentration and a lower ratio of sTfR to ferritin) were associated with increased risk of type 2 diabetes. These associations remained significant after adjustment for a wide range of known risk factors and markers of different pathways of the development of diabetes. Of the individual markers, adjustment for ALT and GGT had the strongest impact on the association observed between ferritin and diabetes risk. In contrast, only a marginal impact on the inverse association between sTfR-to-ferritin ratio and type 2 diabetes was observed in models adjusting for biomarkers of inflammation, hepatic fat accumulation, insulin resistance and dyslipidaemia. Levels of sTfR were not significantly associated with risk of type 2 diabetes.

The results of the present study are consistent with findings from previous prospective studies suggesting that high serum ferritin concentrations [4–6, 13] and lower ratios of sTfR to ferritin [5, 7] are associated with increased risk of type 2 diabetes. Specifically, in a prospective study among Finnish men, Salonen et al [7] observed a 2.5-fold higher risk of diabetes among persons in the lowest compared with the highest quintile of sTfR-to-ferritin ratio; however, their analysis was not adjusted for markers of inflammation, liver enzymes or adiponectin. In the Nurses’ Health Study [5], serum ferritin levels and the sTfR-to-ferritin ratio were associated with an approximately 2.5-fold higher diabetes risk in the highest vs lowest quintile after controlling for C-reactive protein and various conventional risk factors of diabetes. In the EPIC-Norfolk cohort [4], higher ferritin level was associated with diabetes risk after adjustment for conventional risk factors as well as plasma levels of vitamin C, C-reactive protein, fibrinogen, IL-6, liver function tests, ALT, GGT and adiponectin. In contrast to these studies, in the ARIC cohort the association was not significant after adjustment for HDL-cholesterol, waist circumference, hypertension, fasting glucose level and fasting triacylglycerol level [13]. In the present study, we were able to investigate the association of body iron stores, as assessed not only as circulating ferritin concentrations but also as sTfR (and the ratio of sTfR to ferritin), with risk of incident type 2 diabetes in a population-based cohort. In addition, we were able to account for potential confounding effects of a wide range of biomarkers presenting different pathways leading to onset of type 2 diabetes (including hs-CRP, adiponectin, GGT, ALT, fetuin-A, HDL-cholesterol and triacylglycerol), some of which were not accounted for in previous studies. Generally, risk estimates between measures of body iron status and type 2 diabetes were attenuated after inclusion of biomarkers. Exceptions were C-reactive protein and fetuin-A, for which risk estimates increased; however, the increase was small and lay within the limits of uncertainty. We were also able to adjust for circulating HbA_1c_, which, however, as a measure of long-term blood glucose levels, can be considered a rather downstream marker of onset of disease rather than a metabolic pathway to type 2 diabetes. Therefore, we excluded HbA_1c_ levels from the main analysis. Interestingly, though, the relative contributions of the biomarkers were not substantially different when we further adjusted for HbA_1c_ levels in sensitivity analyses.

Two prospective studies that evaluated the association between ferritin and diabetes incidence also measured sTfR levels and reported that not only a high ferritin level but also a low sTfR-to-ferritin ratio was significantly associated with an increased risk of diabetes [5, 7]. However, the independent association between sTfR levels per se and diabetes risk was reported only in one study. That study, conducted within the placebo arm of the Diabetes Prevention Program, suggested that elevated sTfR levels are associated with increased risk of diabetes among overweight and obese persons with impaired glucose tolerance [6]. The authors speculated that sTfR levels may increase as a compensatory mechanism for a reduction in free iron levels that may occur secondary to oxidative stress. Alternatively, the authors suggested that sTfR may be a biomarker of some other factor that is causally related to development of diabetes, possibly unrelated to iron load. In the present study, we found no association between sTfR and diabetes incidence. Interestingly, sTfR tended to be associated with diabetes risk in individuals with HbA_1c_ values below median but not in those with above-median HbA_1c_ values. In the present study circulating levels of sTfR were remarkably lower (average 1.13 mg/l) than those observed in the Diabetes Prevention Program (average 3.4 mg/l). Thus, it is possible that in the present study the contrast between low and high values was too small to reveal an association with increased risk.

The mechanism for the association between ferritin and type 2 diabetes is unknown, but excess iron is usually stored in the liver, muscle and pancreas and may cause organ-specific oxidative stress leading to insulin resistance and beta cell failure. Iron is a catalyst in the formation of hydroxyl radicals, which are highly active and potentially attack cell membrane lipids [27], and subsequent changes in cell membrane may lead to impaired glucose intake of the cell [28, 29]. In the liver, damage due to oxidative stress may cause insulin resistance by interfering with the ability of insulin to suppress hepatic glucose production [30]. The hepatic insulin resistance caused by iron overload seems to be independent of insulin resistance induced by fatty liver disease [31]. In addition, iron accumulation in hepatocytes may interfere with the insulin-extracting capacity of the liver, while iron accumulation also interferes with insulin synthesis and secretion in the pancreas [2]. Hence, iron excess seems to contribute initially to insulin resistance and subsequently to decreased insulin secretion [2]. In addition, iron deposition in muscle has been shown to decrease glucose uptake [32]. Although elevated plasma ferritin levels may be causally related to risk of diabetes, increased ferritin levels may also be a marker of metabolic alterations that may causally increase diabetes risk. For example, increased ferritin values may reflect subclinical systemic inflammation [1, 33], fatty liver [34, 35] or dyslipidaemia [11, 12], which are all potentially related to insulin resistance and risk of type 2 diabetes. However, in the present study the association observed between ferritin and risk of diabetes was independent of biomarkers of inflammation, hepatic fat accumulation, insulin resistance and dyslipidaemia.

Circulating ferritin concentrations are commonly used as an indicator of body iron stores in epidemiological studies. This use, however, is complicated, because ferritin is an acute-phase protein that may be elevated in inflammation, severe liver disease or cancer [8]. Furthermore, its relationship to non-transferring bound iron and the intracellular iron pool, which may be responsible for iron-related oxidative damage, is uncertain [14]. Circulating sTfR concentration has been proposed as a marker of iron status that is less affected by the presence of inflammation. However, low iron stores result in the induction of synthesis of TfRs and they also reflect iron requirement [36]. Therefore, it has been suggested that the ratio of serum concentrations of sTfR and ferritin should be a preferred measure of body iron stores [18, 19, 37]. However, in the present study no notable difference in the strength of the associations with diabetes risk was observed between ferritin and sTfR-to-ferritin ratio. Given that a strong inverse correlation (*r* = −0.97) was observed between ferritin and sTfR-to-ferritin ratio, and no association was observed for sTfR, the association observed for sTfR-to-ferritin ratio is likely to be driven by ferritin only. On the other hand it can be speculated that if sTfR was a more accurate marker of iron stores, then this would indicate that iron stores are not a relevant risk factor for the development of diabetes and that ferritin concentration might reflect something different not fully accounted for in the present–as well as in previous–studies showing a strong association between ferritin and diabetes risk.

All potential cases in our study were verified by a physician. Although we considered only clinically apparent type 2 diabetes and did not screen our study population during follow-up, we excluded participants with plasma glucose values within the diabetic range at baseline, and exclusion of individuals diagnosed within the first 2 years of follow-up did not alter the result. Thus, it is unlikely that prevalent but undiagnosed cases of diabetes have influenced the results. A number of participants were excluded from the original study population for the present analysis. However, excluded cases and non-cases did not differ from those cases and non-cases included in the analysis and we therefore do not expect an influence of exclusion on the risk estimates. We had data available from a single blood withdrawal only, which might have introduced random measurement errors in determining biomarkers. Random measurement error generally tends to bias relative risks in epidemiological studies between biomarkers and disease outcomes towards the null [38]. Also, additional sources of measurement variability (related to blood processing or long-term storage) may have differed between the biomarkers. Thus, we cannot entirely rule out the possibility that some of the above-mentioned limitations may have influenced our results. Although the analyses were adjusted for a large variety of known diabetes risk factors, we cannot rule out the possibility that our observations may have been influenced by other unmeasured factors or by residual confounding due to imprecision in the measurement of covariates.

In conclusion, we found that elevated body iron stores below the levels observed in haemochromatosis are associated with higher risk of type 2 diabetes independently of established risk factors and a range of diabetes biomarkers. However, sTfR levels were not associated with type 2 diabetes and our findings imply that particularly high ferritin levels are related to a higher risk of type 2 diabetes. Further research is warranted to identify predictors of ferritin concentrations, which may help to identify targets for lowering the risk of type 2 diabetes.

## Abbreviations

ALT: Alanine aminotransferase
EPIC: European Prospective Investigation into Cancer and Nutrition
GGT: γ-Glutamyltransferase
hs-CRP: High-sensitivity C-reactive protein
sTfR: Soluble transferrin receptor
TfR: Transferrin receptor
